# *Bordetella petrii* Clinical Isolate

**DOI:** 10.3201/eid1107.050046

**Published:** 2005-07

**Authors:** Norman K. Fry, John Duncan, Henry Malnick, Marina Warner, Andrew J. Smith, Margaret S. Jackson, Ashraf Ayoub

**Affiliations:** * Health Protection Agency, London, United Kingdom;; † Glasgow Dental Hospital and School, Glasgow, United Kingdom

**Keywords:** Bordetella, Bordetella infections, mandibular diseases, antimicrobial drug resistance, ribosomal RNA genes, PCR, ompA, risA

## Abstract

We describe the first clinical isolate of *Bordetella petrii* from a patient with mandibular osteomyelitis. The only previously documented isolation of *B. petrii* occurred after the initial culture of a single strain from an environmental source.

A 67-year-old man visited an emergency dental clinic, where he complained of toothache in the lower right mandibular quadrant. Examination showed a root-filled lower right canine tooth that was mobile and tender to percussion. The tooth was extracted uneventfully under local anesthesia. The patient returned after several days with pain at the extraction site. A localized alveolar osteitis was diagnosed, and local debridement measures were instituted. These measures proved unsuccessful, and repeat examination showed submandibular lymphadenopathy and tenderness to palpation in the buccal sulcus in the extraction site. Radiographs showed no abnormality at this stage.

A course of oral amoxicillin (250 mg, every 8 h for 5 days) followed by oral metronidazole (200 mg every 8 h for 5 days) was prescribed. Symptoms persisted, with increasing severity of pain in the affected area, and the patient was referred to a tertiary referral center. On examination there, the patient had normal full blood count, hematinics, and glucose levels. Ultrasound examination of the submental soft tissue region did not indicate any abnormal pathology. Radiographs showed radiolucencies in the bone surrounding the extraction site. During this period a 3-week course of oral co-amoxiclav (375 mg, every 8 h) was prescribed. A bone biopsy was performed under local anesthesia, and a diagnosis of mandibular osteomyelitis was made.

A portion of the bone biopsy specimen was cultured in 10 mL Fastidious Anaerobe Broth (FAB) (BioConnections, Wetherby, UK) and incubated for 48 h at 37°C in air. After 48 h, the FAB was subcultured onto 1) Fastidious Anaerobe Agar (FAA, BioConnections), incubated for 72 h at 37°C under anaerobic conditions, and 2) Columbia Blood Agar (CBA, BioConnections), incubated for 72 h at 37°C under 5% CO_2_ atmospheric conditions. Culture on both FAA and CBA showed a pure growth of a facultative gram-negative bacillus that had not been identified with routine laboratory protocols. Initial susceptibility testing using disk diffusion indicated apparent susceptibility of the isolate to erythromycin, gentamicin, ceftriaxone, and piperacillin/tazobactam. The isolate was resistant to amoxicillin, co-amoxiclav, tetracycline, clindamycin, ciprofloxacin, and metronidazole. After initial sensitivity results, a 6-week course of oral clarithromycin (500 mg, 8 hourly) was begun.

At follow-up appointments 3 months and 6 months after antimicrobial drug therapy ceased, clinical and radiographic findings were not unusual, and the infected area healed successfully. Despite the successful clinical outcome, the isolate was subsequently shown to be resistant to clarithromycin in vitro (Table). Improvement of the osteomyelitis may also have been facilitated by the biopsy procedure, during which a sequestrum of bone was removed.

**Table:** Results of antimicrobial drug–susceptibility testing for strain GDH030510

Antimicrobial agent	MIC (μg/mL)
Penicillin	>32
Ampicillin	>256
Piperacillin/tazobactam	2
Ceftriaxone	>32
Cefotaxime	>32
Ceftazidime	32
Imipenem	>32
Meropenem	>32
Ertapenem	>32
Amikacin	>256
Gentamicin	4
Tobramycin	16
Ciprofloxacin	>32
Erythromycin	128
Azithromycin	4
Clarithromycin	64
Clindamycin	>256
Chloramphenicol	>256
Cotrimoxazole	8
Rifampin	>32
Tetracycline	>256

The gram-negative bacillus (designated strain GDH030510) was submitted to the Health Protection Agency, Centre for Infections, London, for identification. Preliminary tests results were consistent with those described for members of the genus *Bordetella*. Colonies had the following phenotypic characteristics: positive reaction for oxidase and negative reaction for urease production, motility using the hanging-drop method at 37°C, and slide agglutination with *B. pertussis* and *B. parapertussis* antiserum (Difco, Shannon, Ireland). The organism could be cultured on MacConkey agar and was nonhemolytic on blood agar. Genomic DNA was extracted by using the InstaGene Purification Matrix (BioRad, Hercules, CA, USA). DNA amplification of small-subunit (SSU) rRNA genes was performed by using primers 27f and 1525r (1). Amplification and sequencing of the gene for the *Bordetella* outer membrane protein A (*ompA*) and the RisA response regulator (*risA*) were as described by von Wintzingerode et al. (2). Reaction mixes contained the following: 2 mmol/L MgCl_2_, 10 mmol/L Tris-HCl (pH 8.3), 50 mmol/L KCl, 0.05% W-1 (Invitrogen, Paisley, UK), 0.2 μmol/L of each deoxynucleotide (Roche Applied Science, Lewes, UK) 20 pmol of each primer (MWG Biotech, Milton Keynes, UK), 2.5 U of Taq DNA polymerase (Invitrogen), 1.0 mol/L betaine (Sigma-Aldrich, Gillingham, UK), and 10 μL template DNA. Amplification was performed in a DNA Engine (MJ Research, Bio-Rad) by using 35 cycles of denaturation of 1 min at 94°C, 1 min at 55°C, and 2 min at 72°C and a final step at 72°C for 3 min. Amplicons from triplicate samples were pooled and purified with Montage PCR96 filter plates (Millipore, Watford, UK). For the rRNA gene, nucleotide sequence was determined by using the primers used for amplification, together with internal primers (1). Sequencing was performed with the Dye Terminator Cycle Sequencing kit (Beckman Coulter, High Wycombe, UK) and analyzed on a CEQ 8000 Genetic Analysis System (Beckman Coulter), according to the manufacturer's instructions. Contig assembly and sequence analyses were performed with Kodon, version 2.0 (Applied Maths, Kortrijk, Belgium) and BioNumerics, version 3.5 (Applied Maths). Consensus sequence was compared with public databases with the BLASTn program (http://www.ncbi.nlm.nih.gov/BLAST) (3), and sequences with the greatest similarity were downloaded for further analysis.

The nucleotide sequences of the 16S rRNA gene, *risA* gene, and *ompA* gene of strain GDH030510 have been submitted to the EMBL Nucleotide Sequence Database under accession numbers AJ870969, AJ920265, and AJ920264, respectively. The 1,486 nucleotides (nt) of the SSU rRNA gene sequence that were determined showed a maximum similarity of 99.3% (1,468/1,479 nt) with the 16S rRNA gene from the type strain of *B. petrii* (GenBank accession no. AJ249861). For all sequence analyses, regions not present in all sequences and ambiguous bases were excluded. The species with the next highest similarities were *B. parapertussis*, 98.4% (1,455/1,479 nt), and *B. bronchiseptica*, 98.3% (1,454/1,479 nt). The secondary structure of the SSU rRNA from both strain GDH030510 and the submitted sequence for the type strain of *B. petrii* were compared to that proposed for bacteria (4). Where possible (i.e., in stems), supportive evidence from base-pairing was sought. This analysis indicated that the assigned nucleotides at 3 separate locations in the GenBank *B. petrii* sequence were not supported, as they formed noncanonical base-pairing (C-U vs. C-G [156, 165], U-U vs. A-U [824, 875], A-C vs. G-C [838, 848]; *Escherichia coli* numbering [5]). Exclusion of these 3 bases increased the similarity to 99.5% (1,468/1,476 nt). The 445 nt of the *risA* gene sequence of strain GDH030510 that were determined showed a maximum similarity value of 93.9% (418/445 nt) with the *risA* gene from the *B. petrii* type strain (AJ242553). The species with the next highest similarities were *B. parapertussis*, *B. avium*, *B. bronchiseptica*, and *B. pertussis*, all with similarities of 88.3% (393/445 nt). The 414 nt of the *ompA* gene sequence that were determined showed maximum a similarity value of 92.0% (381/414 nt) with the *ompA* gene from the *B. petrii* type strain (AJ242599). The species with the next highest similarities were *B. bronchiseptica* and *B. parapertussis*, both with similarities of 87.9% (364/414 nt).

Further susceptibility testing was undertaken, with MICs determined by agar dilution on diagnostic sensitivity test agar (Oxoid, Basingstoke, UK) supplemented with 5% lysed horse blood, with inocula equivalent to 0.5 and 2 McFarland standards, and with incubation at 37°C in air for 24 to 36 h to ensure adequate growth. No inoculum effects were noted on MICs for any antimicrobial agents. MICs are shown in the Table.

Currently, the genus *Bordetella* comprises 8 species (2), 7 of which have been isolated from humans and a variety of warm-blooded animals. Three of these species, *B. pertussis*, *B. parapertussis*, and *B. bronchiseptica*, are often referred to as the classic *Bordetella* species (6); they are closely related phylogenetically but have distinct host ranges. *B. pertussis* is an obligate pathogen for humans and is the etiologic agent of pertussis. *B. parapertussis* causes a similar, usually milder, infection in humans, and *B. parapertussis* strains may also be isolated from sheep with chronic pneumonia. Human isolates of *B. parapertussis* are highly clonal and appear distinct from sheep isolates. *B. bronchiseptica* is known to infect many mammals, including humans, although human infection is rare and usually occurs in immunocompromised hosts. *B. avium* is pathogenic for birds, including poultry, and causes coryza or rhinotracheitis in turkeys. Three other species of *Bordetella*, *B. hinzii*, *B. holmesii*, and *B. trematum*, have subsequently been described (7–9). *B. hinzii* is a commensal of the respiratory tract of fowl but has some pathogenic potential in immunocompromised humans (10) and was implicated as the causative agent in a fatal case of septicemia (11). *B. holmesii* has been isolated from blood of young adults, and nasopharyngeal specimens (9,12,13). *B. trematum* has been isolated from human ear infections and wounds, but its pathogenicity remains unknown (7). The most recently recognized species, *B. petrii*, was described in 2001 (2) and was isolated from an anaerobic bioreactor culture enriched from river sediment. This description was based on a single isolate and, to our knowledge, no further isolates of this species have been previously reported from any source.

The source of infection of the strain described here and the pathogenic role of *B. petrii* are currently unknown. However, the identification and characterization of further clinical isolates should help determine the reservoir and virulence potential of this intriguing species.

## Addendum

A clinical isolate of a new species of *Bordetella*, *Bordetella ansorpii* sp. nov, has recently been reported (14). Phylogenetically, it appears to be more closely related to *B. petrii* than to other members of the genus.
